# Being Married Increases Life Expectancy of White but Not Black Americans 

**Published:** 2019-09

**Authors:** Shervin Assari, Mohsen Bazargan

**Affiliations:** 1 Departments of Family Medicine, College of Medicine, Charles R Drew University of Medicine and Science, CA, United States; 2 Departments of Family Medicine, University of California, Los Angeles (UCLA), Los Angeles, CA, United States

**Keywords:** Population Differences, Population Groups, Ethnicity, Ethnic Groups, Blacks, African Americans, Chronic Medical Conditions, Chronic Disease, Socioeconomic Status, Socioeconomic Position, Marital Status, Family Type

## Abstract

**Objective:** The positive effect of high socioeconomic position (SEP) on health is well established. According Minorities’ Diminished Returns (MDRs) theory, however, the SEP-health link is smaller for Blacks compared to Whites. Using a 25-year follow up data of a national sample, this study tested racial differences in the effects of marital status on life expectancy among American adults.

**Materials and methods:** The data of Americans’ Changing Lives (ACL, 1986 – 2011) were used. The ACL is a nationally representative longitudinal cohort study followed 3,361 White or Blacks adults from 1986 to 2011. The predictor of interest was marital status in 1986. Confounders included demographic factors (age and gender), SEP (education and employment), health behaviors (drinking, smoking, and physical activity), and health status (depressive symptoms, chronic disease, and self-rated health) all measured at baseline. Race was the moderator variable. All-cause mortality was the main dependent variable (outcome). Cox proportional hazard modeling was applied for data analysis.

**Results:** In the overall sample, individuals who were married at baseline had a lower risk of mortality during the 25 years of follow up. Race altered the effect of marital status on life expectancy, indicating smaller protective effect for Blacks relative to Whites. Race –specific Cox regression models showed an association between marital status and life expectancy for White but not Black Americans.

**Conclusion:** In line with the MDRs theory, the health gain that follows marital status is diminished for Black Americans compared to White Americans. Only equalizing SEP across racial groups may not be adequate for eliminating racial/ethnic health inequalities. Policies should go beyond SEP and reduce societal and structural barriers that disproportionately hinder Blacks from translating their SEP indicators to desirable health outcomes.

## Introduction

The health effect of socioeconomic position (SEP) indicators is enduring, consistent, and growing (1, 2). Almost 

all longitudinal studies in US and Europe have document a positive association between high SEP (e.g. education, income, and family type) and population health (3-5). Availability of SEP resources is one of the main factors that shape social gradient of health and illness (3-5). With a traditional assumption that one size may fit all, however, most research on the health effects of SEP has ignored how sub-populations differ in the effects of SEP on health (3-5). 

Although SEP indicators have overall effects that are positive (3-5), sub-populations may vastly differ in the health gain that follows high SEP (6-11). That is, sub-populations do not similarly translate their SEP resources to tangible health outcomes (10, 12-17). Although high SEP reduces exposure to risk and increase availability of buffers when risk occurs, these effects differ across population groups (9, 18-20). High SEP does not similarly reduce exposures to environmental risk factors across all social groups (11, 20). For example, highly educated Blacks are more likely than highly educated Whites to be exposed to second hand exposure to tobacco smoke (11). Different from the overall health effects of SEP (3, 5, 21, 22), there is a growing literature that documents that the operating mechanism as well as the size of the effects of SEP indicators are not invariant across populations (23-26). Context and life conditions differ across demographic groups which in turn results in differences in how SEP translates to health (27). Due to racism, populations are treated differently by the society, and SEP differentially provides access to opportunity structures for social groups. As a result, the health gain that is expected to follow the very same SEP indicators in unequal across demographic and racial groups (8, 12, 14, 15, 18, 19, 28).

When compared to Blacks, high SEP better increases material resources and assets (18, 19) for Whites than Blacks. Upward social mobility that should promote health increases the psychological costs of high SEP for Blacks (19, 20, 29). Education attainment has smaller effects on health behaviors (16, 30) as well as human capital (31) among Black in comparison to Whit Americans. Thus, racial/ethnic disparities remain despite across all SEP levels. As explained by the Minorities’ Diminished Return (MDR) Theory (32, 33), health gains that follow high SEP is systemically smaller for racial and ethnic minorities. It has been shown that SEP indicators such as education and income have smaller effects on health behaviors such as suicide (8, 34), anxiety (13), depression (35, 36), smoking (16, 37), drinking (38), diet (39), obesity (40, 41) , chronic diseases (17, 42), and mortality (43) of Blacks and Hispanics than Whites.

As a result of MDRs (7, 33), high SEP Blacks show worse than expected health across domains (13, 31, 32, 40, 44). This is a challenge because it challenges the health and well-being of middle-class Blacks (9, 18, 45). and Blacks gain less health from their education attainment, income, employment, and marital status. Less, however, is known on racial / ethnic group differences in the effect of marital status on mortality. 

***Aims:*** To investigate whether or not Minorities’ Diminished Returns also holds for the effects of marital status on life expectancy of adults, this study used long term follow up data of a national sample of American adults to explore the Black-White differences for the effect of marital status at baseline on the risk of all-cause mortality over time. In line with other studies that have documented Blacks’ diminished returns (44, 46), and those studies that have documented poor health of high SEP Blacks (6, 15, 47, 48), we expected smaller effect of being married on delaying mortality for Blacks than Whites.

## Materials and methods

***Design and Setting: ***The Americans’ Changing Lives (ACL), 1986– 2011, is a longitudinal study with a nationally-representative sample of American adults. Detailed information on the ACL methodology, sampling, and data collection is presented elsewhere (49, 50).

***Participants and Sampling: ***The ACL sampling was a stratified / multistage / probability sampling. ACL sampling included an oversampling of older American adults (age > 60 years) and Black Americans Participants were 3,617 adults (age of 25 years or older) who were all non-institutionalized US adults who were residing in the continental US in 1986. Current analysis is limited to 2205 on-Hispanic Whites and 1156 non-Hispanic Blacks (n = 3,361).

***Process: ***ACL baseline data collection used a face-to-face interview mode. Other data were collected from National Death Index (NDI) and Death certificates. 

***Study Measures*** *:* *Demographic Characteristics: *Demographic factors included gender (male as the referent category) and age (an interval variable).

*Socioeconomic position (SEP): *Two SEP confounders in the current study were employment status and educational attainment (years of schooling), both collected at baseline in 1986. While education attainment was treated as a continuous variable, employment status was a dichotomous variable (1 employed, 0 unemployed).

*Race/Ethnicity: *Race was the effect modifier, defined as Blacks = 1 versus Whites = 0. Data on participant’s race were gathers at wave 1 (1986) using multiple questions. Participants Hispanic origin were assessed using an open-ended question. Respondents were asked “In addition to being American, what do you think of as your ethnic background or origins?” and “Are you white, black, American Indian, Asian, or another race?”. Multiple answers were allowed. Individuals who self-identified with more than one race/ethnic groups were asked to identify the race/ethnicity which best describes them. These items were used to construct the race/ethnicity variable: “Non-Hispanic White” and “Non-Hispanic Black”.

*Depressive Symptoms: *Frequency of symptoms of depression were evaluated using the 11-item Center for Epidemiological Studies-Depression scale (CES-D) (51-53). CESD questionnaire measures the extent to which a respondent has felt depressed, sad, lonely, and restless over the past month. The abbreviated CES-D scale is reliable and valid (54). Item responses vary between 1 (never or hardly ever) to 3 (most of the time), resulting in a CES-D score ranging from 11 to 33. Higher CES-D score indicates greater frequency of depressive symptoms.

*Chronic Disease (CD): *Number of CD was assessed using self-reported diagnoses made by a health care provider. Participants provided information regarding whether health care providers have ever told them they have stroke, diabetes, cancer, heart disease, hypertension, arthritis, and lung disease. A total CD score was summed, resulting in a numerical scale ranging between 0 and 7 (55).

*Self-Rated Health (SRH). *Respondents classified their own overall health status from excellent (score 1) to poor (score 5). We treated our SRH variable as a continuous measure with a potential range from 1 to 5, where a higher score indicating worse health status (56).

*All-Cause Mortality: *Data on time and cause of death from mid-1986 through 2011 were obtained through death certificates, National Death Index (NDI) or informants. In almost all cases, time and cause of death could be verified using a death certificate. Mortality information were collected regardless of the participants’ follow up status in the cohort study. That is, all participants were evaluated for mortality, even if they were dropouts or loss to follow ups. Overall, 1,737 Black and White participants were deceased, while 1,624 Black and White individuals survived the 25 year follow up study. 

***Statistical analysis: ***To adjust for the complex sampling design of the ACL, Stata 15.0 was applied for data analysis. Using Stata allowed us to estimate all standard errors (SEs) based on the sampling weights. Taylor series linearization method was used for estimating designed based SEs. Four Cox proportional hazards models were estimated for multi-variable modeling. Cox proportional hazard models require two distinct outcomes: 1) a binary event outcome (mortality), and 2) time to even (mortality) since baseline. Mortality was coded as 1 for individuals whose death occurred anytime from 1986 to 2011. Time of death (or censoring), was calculated in months from baseline to the time of death or the year 2011. To test the Cox proportional hazard models’ assumptions, we conducted Schoenfeld residual analysis using estat phtest in Stata.

The independent variable was marital status at baseline measured in 1986, while the outcomes were time to all-cause mortality during the 25 year follow up period. Confounding variables included baseline demographics (age, gender), socio-economic characteristics (education, employment), health behaviors (alcohol drinking, cigarettes smoking, and physical activity), and health status (CD, SRH, and depressive symptoms) all measured at baseline in 1986. Race was the main moderator. 

First, we estimated a model in the pooled sample to estimate the main effect of being married on the risk of all-cause mortality, net of covariates. To test if race/ethnicity and marital status interact on mortality risk, we ran the Model 2 with an interaction term between race/ethnicity and marital status. Finally, we ran race/ethnicity -stratified models. Hazard ratios (HRs), SEs, 95% Confidence Intervals (CIs), as well as *p *values were reported.

## Results

***Descriptive Statistics:*** Table 1 presents descriptive statistics for all study variables used in the overall sample and by race. Age and gender were not significantly different between White and Black Americans. Black individuals had lower education and employment status and worse health status (CD, SRH, and depressive symptoms) compared to White individuals. Blacks had lower life expectancy compared to Whites.

***Pooled Model:*** Table 2 presents the results for two Cox proportional hazards models in the pooled sample. The results of *Model 1* show a significantly lower all-cause mortality risk for those who were married (HR = 0.84, p = 0.016), net of confounders. *Model 2*indicated an interaction between race/ethnicity and marital status (HR = 1.29, p = 0.048), net of covariates, suggesting that the health gain associated with being married is smaller for Blacks than Whites.

**Table 1:** Descriptive Statistics Overall and By Race / Ethnicity

**All**		**Whites**		**Blacks**		
	**Mean (SE)**	**95% CI**	**Mean (SE)**	**95% CI**	**Mean (SE)**	**95% CI**
Age	47.77 (.534)	46.69 – 48.84	47.96 (.601)	46.75 – 49.17	46.33 (.717)	44.89 – 47.78
Education*	12.53 (.096)	12.34 – 12.73	12.69 (.105)	12.48 – 12.90	11.37 (.233)	10.90 – 11.84
Depressive symptoms*	−0.03 (.025)	−0.08 – 0.02	−0.07 (.025)	−0.13 – 0.02	0.28 (.051)	0.18 – 0.38
Chronic Disease*	0.79 (.028)	0.74 – 0.85	0.78 (.031)	0.71 – 0.84	0.91 (.052)	0.81 – 1.02
	**% (SE)**	**95% CI**	**% (SE)**	**95% CI**	**% (SE)**	**95% CI**
Gender						
Men	47.26 (.012)	44.86 – 49.68	47.82 (.013)	45.12 – 50.52	43.18 (.022)	38.79 – 47.69
Women	52.74 (.012)	50.32 – 55.14	52.18 (.013)	49.48 – 54.88	56.82 (.022)	52.31 – 61.21

* Significantly different between White and Black Americans.

***Race stratified Models:*** Table 3 shows the results of *Model 3* and *Model 4* that represent Cox regression models for Whites and Blacks respectively. Results from *Model 3* and *Model 4* show that being married was significantly related to lower mortality risk among White Americans (HR = 0.81, p = 0.004), while this association is not significant for Black Americans (HR = 0.98, p = 0.869).

**Table 2:** Summary of Cox Regressions in the Pooled Sample

	**HR**	**SE**	**95% CI**		**z**	**P**
Model 1						
Marital Status (Married)	0.84	0.06	0.73	0.97	-2.51	0.016
Race (Blacks)	1.10	0.08	0.94	1.28	1.22	0.229
Gender (Female)	0.51	0.03	0.44	0.58	-9.83	0.000
Age	1.08	0.00	1.07	1.09	23.26	0.000
Education	0.98	0.01	0.96	1.00	-1.86	0.069
Employment Status (Employed)	0.69	0.07	0.56	0.84	-3.77	0.000
Smoking (Smoker)	1.80	0.14	1.55	2.10	7.73	0.000
Drinking	1.00	0.06	0.88	1.14	0.02	0.981
Physical Activity	0.88	0.02	0.83	0.93	-4.51	0.000
Obese	1.03	0.08	0.88	1.22	0.41	0.686
Self-Rated Health (Poor)	1.20	0.05	1.10	1.31	4.27	0.000
Depressive Symptoms	0.99	0.04	0.92	1.08	-0.13	0.894
Chronic Disease (CD)	1.12	0.03	1.06	1.18	4.07	0.000
Model 2						
Marital Status (Married)	0.81	0.06	0.70	0.94	-2.95	0.005
Race (Blacks)	0.97	0.08	0.81	1.15	-0.40	0.690
Gender (Female)	0.51	0.04	0.44	0.59	-9.77	0.000
Age	1.08	0.00	1.07	1.09	23.16	0.000
Education	0.98	0.01	0.96	1.00	-1.89	0.065
Employment Status (Employed)	0.69	0.07	0.56	0.84	-3.80	0.000
Smoking (Smoker)	1.80	0.14	1.54	2.10	7.70	0.000
Drinking	1.00	0.06	0.88	1.14	0.04	0.970
Physical Activity	0.88	0.02	0.83	0.93	-4.44	0.000
Obese	1.03	0.08	0.88	1.22	0.39	0.697
Self-Rated Health (Poor)	1.20	0.05	1.10	1.31	4.32	0.000
Depressive Symptoms	0.99	0.04	0.92	1.07	-0.16	0.875
Chronic Disease (CD)	1.12	0.03	1.06	1.18	4.12	0.000
Marital Status (Married)* Race (Blacks)	1.29	0.16	1.00	1.66	2.03	0.048

Source: Americans’ Changing Lives (ACL), 1986 – 2011. Sample size: 3,361 White and Black adults. Outcome: All-cause mortality between 1986 and 2011. Hazard ratios (HR), Standard Errors (SE), 95% Confidence Intervals (95% CI)

**Table 3:** Summary of Cox Regressions in White and Black Americans

	**HR**	**SE**	**95% CI**		**z**	**p**
Model 3 (Whites)						
Marital Status (Married)	0.81	0.06	0.70	0.93	-3.00	0.004
Gender (Female)	0.48	0.04	0.41	0.56	-9.36	0.000
Age	1.09	0.00	1.08	1.09	20.67	0.000
Education	0.98	0.01	0.95	1.00	-1.99	0.053
Employment Status (Employed)	0.70	0.08	0.56	0.87	-3.28	0.002
Smoking (Smoker)	1.85	0.17	1.54	2.23	6.66	0.000
Drinking	1.02	0.07	0.88	1.18	0.27	0.791
Physical Activity	0.88	0.03	0.82	0.94	-4.12	0.000
Obese	1.01	0.10	0.82	1.23	0.05	0.960
Self-Rated Health (Poor)	1.25	0.06	1.13	1.37	4.64	0.000
Depressive Symptoms	0.99	0.05	0.90	1.09	-0.20	0.840
Chronic Disease (CD)	1.13	0.03	1.06	1.20	4.08	0.000
Model 3 (Blacks)						
Marital Status (Married)	0.98	0.11	0.78	1.24	-0.17	0.869
Gender (Female)	0.70	0.08	0.55	0.88	-3.16	0.003
Age	1.06	0.01	1.05	1.08	11.26	0.000
Education	0.98	0.01	0.96	1.01	-1.52	0.135
Employment Status (Employed)	0.66	0.12	0.46	0.96	-2.27	0.029
Smoking (Smoker)	1.53	0.15	1.26	1.87	4.37	0.000
Drinking	0.98	0.14	0.74	1.31	-0.13	0.899
Physical Activity	0.89	0.05	0.79	1.00	-1.98	0.054
Obese	1.12	0.13	0.88	1.42	0.93	0.357
Self-Rated Health (Poor)	1.03	0.05	0.94	1.12	0.57	0.571
Depressive Symptoms	0.95	0.04	0.88	1.04	-1.17	0.249
Chronic Disease (CD)	1.09	0.05	0.99	1.20	1.78	0.083

Source: Americans’ Changing Lives (ACL), 1986 – 2011. Sample size: 3,361 White and Black adults. Outcome: All-cause mortality between 1986 and 2011. Hazard ratios (HR), Standard Errors (SE), 95% Confidence Intervals (95% CI)

## Discussion

Blacks and Whites differ in the protective effect of marital status on risk of mortality over 25 years. White Americans, but not Black Americans, show a reduction in their risk of all-cause mortality due to being married. 

Racial and ethnic differences in the link between family type/marital status are in line with Minorities’ Diminished Return theory (7, 33), which suggests SEP indicators better serve health of White than Black Americans. For instance, the effects of education, income, and family type on suicide(8, 34), anxiety(13), depression (35, 36), smoking (16, 37), drinking(38), diet(39), obesity (40, 41) , chronic diseases (17, 42), and mortality (43) are smaller for Blacks than Whites. This study, however, extends the literature on Minorities’ Diminished Return (MDR) theory (7, 33) by showing that the life expectancy gain from marital status is smaller for Blacks than for Whites.

Race (28), gender (57), and their intersection (47) alter health gains that follow SEP (1, 2, 58, 59). One extreme example of the minorities’ diminished return is poor mental health of high SEP Black men and women (28, 57). High education and high-income Black men are at higher risks of depressive symptoms and clinical depression (36, 47). 

There has been a tendency to blame Blacks poor health to their behaviors such as family type. This study questions such attitude by documenting that family type is not the reason why SEP is less protective for Blacks than Whites (44, 46, 60, 61). Marital status, itself, generates smaller health effects for Whites than Blacks (13). This is illuminating and suggests that lower marriage rate is not a source of disparities for Blacks. We argue that family type is not a cause but a consequence of racial inequalities in the US. 

Societal rather than biological mechanisms should be investigated to explain why SEP better promote health of Whites than Blacks. Society treats Blacks worse than Whites across SEP levels, so the protective effects of same SEP indicator would be smaller for Blacks than Whites. Due to residential segregation, labor market discrimination, low quality of education, and other aspects of racism, Black families may have difficulty competing with their White American counterparts who more easily can secure low stress, high paying, prestigious jobs. All these societal processes may have a role and need more research (19, 20, 45, 62).

Due to racism and discrimination as well as unfair treatment by the society, upward social mobility is more costly Blacks in comparison to Whites (29, 45). As a result, high SEP Blacks turn to high effort coping to deal with stress (63, 64). John Henryism, an effortful coping which induces several health hazards, is common among high SEP Blacks (65-67). High SEP Blacks also report high levels of goal striving stress, which is linked to health problems (68-72). At an individual level, these phenomena increase psychological costs of upward social mobility for Black Americans (18-20, 29), however, these are probably adjustments to cope with unfair treatment by the society.

## Conclusion

To conclude, Blacks and Whites differ in the health effects of marital status in terms of life expectancy gain over 25 years. Blacks and Whites do not equally benefit from their SEP indicators, with Blacks being in a relative disadvantage when compared to White Americans, possibly because of structural racism and interpersonal discrimination. Future research should study the exact societal and structural barriers that may hinder Blacks from translating their available SEP indicators to tangible outcomes. There might be also a need to find the economic and public policies that can effectively undo Blacks’ diminished return of SEP. Policy makers should be aware that policy and programs that increase SEP may have differential health effects on various populations.
